# Use of Real-Time Continuous Glucose Monitoring Improves Glycemic Control and Other Clinical Outcomes in Type 2 Diabetes Patients Treated with Less Intensive Therapy

**DOI:** 10.1089/dia.2021.0212

**Published:** 2022-01-05

**Authors:** Thomas Grace, Jay Salyer

**Affiliations:** Endocrinology & Diabetes Specialists of Northwest Ohio, Findlay, Ohio, USA.

**Keywords:** Type 2 diabetes, CGM, HbA1c, Time in range, Basal insulin, Noninsulin medications

## Abstract

***Objective:*** Use of real-time continuous glucose monitoring (rtCGM) has been shown to improve glycemic control in patients with type 2 diabetes (T2D) who are treated with intensive insulin therapy. However, most T2D patients are denied coverage for rtCGM due to failure to meet payer eligibility requirements: treatment with ≥3 insulin injections (or pump) and history of 4 × /day blood glucose testing. We investigated the relevance of these criteria to successful rtCGM use.

***Methods:*** This 6-month, prospective, interventional, single-arm study assessed the clinical effects of use rtCGM in patients with T2D treated with basal insulin only or noninsulin therapy. Primary outcomes were changes in HbA1c, average glucose, glycemic variability (% coefficient of variation), and percent of time in range (%TIR), below range (%TBR) and above range (%TAR).

***Results:*** Thirty-eight patients were included in the analysis (10.1% ± 1.8% HbA1c, 54.7 ± 10.2 years, 35.6 ± 6.4 body mass index). At 6 months, we observed reductions in HbA1c (−3.0% ± 1.3%, *P* < 0.001) and average glucose (−23.6 ± 38.8, *P* < 0.001). %TIR increased 15.2 ± 22.3, from 57.0 ± 29.9 to 72.2 ± 23.6, *P* < 0.001, with all patients maintaining %TBR targets (<4% at 70 mg/dL, <1% at <54 mg/dL). No changes in glycemic variability were observed. The greatest improvements in %TIR and %TAR were seen in patients treated with ≤1 medication.

***Conclusions:*** rtCGM use was associated with significant glycemic improvements in T2D patients treated with basal insulin only or noninsulin therapy. Given the growing body of evidence supporting rtCGM use in this population, insurance eligibility criteria should be modified to expand rtCGM use by T2D patients treated with less intensive therapies.

## Introduction

Suboptimal glycemic control persists among a substantial percentage of individuals with type 2 diabetes (T2D).^1,2^ The most recent data show that the percentage of individuals who achieved their glycemic targets declined from 69.8% in 2010 to 63.8% in 2014; whereas, the percentage of those with HbA1c >9.0% (>75 mmol/mol) increased from 12.6% to 15.5% during the same time period.^1^ Although use of blood glucose monitoring (BGM) remains common in insulin-treated and noninsulin-treated T2D patients, studies have not consistently shown efficacy in using BGM to change patient behavior or reduce HbA1c in the noninsulin-treated population.^3^

Randomized controlled trials (RCTs) have demonstrated that use of real-time continuous glucose monitoring (rtCGM) reduces HbA1c levels and confers other glycemic benefits in individuals with T2D treated with intensive insulin regimens.^4–6^ Although intermittent and short-term rtCGM use in T2D patients who are treated with less intensive therapies has also shown similar benefits,^7–10^ the clinical efficacy of routine rtCGM use in this population has not been well studied.

However, studies have shown that T2D patients treated with basal insulin only or antihyperglycemic medications (e.g., sulphonylureas) are at increased risk for frequent and/or severe hypoglycemia, particularly older patients.^11–13^ In addition to the clinical consequences of severe hypoglycemia, these episodes have been shown to diminish patient's willingness to adhere to their prescribed treatment regimens, which can lead to deterioration of glycemic control and increased risk of long-term complications.^14,15^

Despite a growing body of evidence supporting the potential benefits of rtCGM in managing less intensive diabetes treatment regimens, many government payers (e.g., state Medicaid programs) and most private health plans currently require treatment with intensive insulin management (multiple daily insulin injections or insulin pump) for rtCGM coverage. Thus, the majority of T2D patients are denied access to this technology unless they are willing to pay out-of-pocket.^16^

We report findings from a single-arm study of rtCGM use within cohort of adult T2D patients treated with basal insulin only or noninsulin therapies.

## Methods

### Design and participants

This investigator-initiated, open-label, prospective, interventional, single-arm study assessed the clinical effects of rtCGM use over 6 months in patients with T2D treated with basal insulin only or noninsulin therapies. The primary objective of the study was to evaluate health outcomes, including changes in glycemic control, weight, and body mass index (BMI) after 3 and 6 months of rtCGM use. The study was conducted at the Blanchard Valley Diabetes Center (BVDC), Findlay, OH, from March 10, 2020 through December 31, 2020.

The study was conducted in accordance with Good Clinical Practice, in accordance with the United States Code of Federal Regulations, Title 21, Part 50 (21CFR50).^17^ The study protocol was approved by a central Institutional Review Board (Blanchard Valley Hospital). All participants provided written informed consent before enrollment in the study. Inclusion criteria were as follows: diagnosed T2D, HbA1c >7.5% (>58 mmol/mol), and treatment with basal insulin, noninsulin injectable antidiabetic medication, oral antidiabetic medication, and/or diet and exercise. Exclusion criteria were the following: diagnosed T1D, HbA1c ≤7.5% (≤58 mmol/mol), or treatment with prandial or premix insulin.

### Procedures

Over the 6-month study period, patient visits occurring at initial screening and baseline, followed by clinic visits at months 3 and 6. At screening, demographic information, medical history, information about current medications, and relevant biometric measurements (weight, BMI) were obtained and documented. Blood samples were drawn for HbA1c levels.

At the baseline visit, eligibility was confirmed, and investigators obtained written consent from all patients enrolled in the study. Patients received their rtCGM device (Dexcom G6; Dexcom, Inc., San Diego, CA) and were provided comprehensive training in device use and data interpretation. Alarms/alerts were discussed, and settings were individualized for each patient.

At the 3- and 6-month visits, patients brought their rtCGM device to the BVDC where rtCGM data were downloaded and reviewed by investigators and patients. Treatment changes were initiated if any problematic glycemia or other issues were observed. All adverse events were documented. Blood samples for HbA1c measurement were obtained and biometric measurements documented.

### Outcomes

The primary outcome was change in relevant glycemic metrics from baseline at 6 months. Metrics included HbA1c; average glucose; Glucose Management Indicator value; percentages of time in range (%TIR, 70–180 mg/dL), time below ranges (%TBR, <70 mg/dL, <54 mg/dL), time above ranges (%TAR, >180 mg/dL, >250 mg/dL), and glycemic variability (assessed as coefficient of variation [%CV]). The most recent HbA1c value obtained within the previous 6 months served as the baseline measure for this metric. rtCGM data obtained during the first month of use service as the baseline values for these metrics. Secondary outcomes included changes in body weight and BMI.

### Measures

HbA1c was measured at a local laboratory. All other glycemic measures were obtained from rtCGM data as interpreted in the Dexcom Clarity data management software. Biometric measurements were obtained at the BVDC. For all rtCGM metrics, data from the first month of rtCGM use were utilized as surrogate baseline for assessment of changes.

### Statistical analysis

Basic descriptive statistics, including means, standard deviations, ranges, and percentages, were used to characterize the study participants. Changes in glycemic parameters were reported on all patients and stratified by therapy groups: ≤1 medication (insulin, noninsulin, diet/exercise) and ≥2 Medications (insulin, noninsulin). Two-sided Wilcoxon signed rank tests were used to compare differences in HbA1C, %TIR, %TAR, average glucose and %CV endpoints at baseline and after 3 and 6 months, and weight and BMI were compared at baseline and after 6 months. All tests used a 0.05 significance level, and all computations were performed using SAS^®^ software, version 9.4 (SAS Institute, Inc., Cary, NC).

## Results

Among the 43 patients enrolled in the study, 38 had complete HbA1c for primary outcome analysis. Patients not included in our analyses included 2 patients who withdrew consent, 1 who died from COVID-19 infection, 1 who had insufficient data due to switching to professional rtCGM after the study, and 1 who had no 6-month HbA1c test results.

The cohort was predominantly Caucasian and overweight/obese with suboptimal glycemic control. Gender proportions were well balanced. Most patients had diagnosed T2D for ∼10 years (Table 1). All patients were performing BGM <3 times per day before enrollment. Most patients were treated with metformin and/or an antihyperglycemic medication (sulfonylurea/meglitinide or basal insulin). During the 6-month study period, therapy was intensified in 20 patients and 15 patients had their medications changed or reduced. No severe hypoglycemic events or device-related adverse events occurred during the study period.

**Table 1.** Baseline Characteristics

Characteristics	Overall (*N* = 38)
Age, years (mean ± SD)	54.7 ± 10.2
Gender, *n* (%)	
Female	18 (47.4)
Male	20 (52.6)
Race, *n* (%)	
Caucasian	37 (97.4)
Hispanic	1 (2.6)
Weight, kg (mean ± SD)	103.5 ± 18.2
Body mass index, kg/m^2^ (mean ± SD)	35.6 ± 6.4
Diabetes duration, years (mean ± SD)	13.9 ± 8.8
HbA1C, % (mean ± SD)	10.1 ± 1.8
% Time in range (70–180 mg/dL) (mean ±SD)	57.0 ± 29.9
% Time above range (>180 mg/dL)^a^ (mean ± SD)	43.1 ± 30.5
% Time above range (>250 mg/dL)^a^ (mean ± SD)	15.8 ± 22.9
Average glucose, mg/dL (mean ± SD)	180.1 ± 47.3
% Glucose CV^b^ (mean ± SD)	24.6 ± 5.9
Medications	
Sulfonylurea, *n* (%)	15 (39)
Metformin, *n* (%)	21 (55)
Thiazolidinedione, *n* (%)	5 (13)
DPP-4 Inhibitor, *n* (%)	12 (32)
GLP-1 agnonist, *n* (%)	9 (24)
SGLT-2 inhibitor, n (%)	2 (5)
Basal insulin, *n* (%)	16 (42)

^a^ Two subjects' % time above range (>180 mg/dL), % time above range (>250 mg/dL) at baseline were missing.

^b^ Three subjects' %CV at baseline were missing.

CV, coefficient of variation; SD, standard deviation.

### HbA1c

**FIG. 1.**
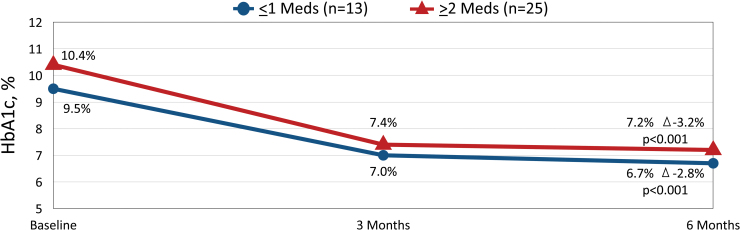
Changes in HbA1c at 3 and 6 months by Therapy Group.

Within the full cohort, we observed a significant reduction in mean HbA1c from baseline to 3 months (10.1% ± 1.8% to 7.3% ± 1.3%, Δ −2.8% ± 1.4%, *P* < 0.001) and 6 months (10.1% ± 1.8% to 7.1% ± 1.2%, Δ −3.0% ± 1.3%, *P* < 0.001). Similar HbA1c reductions were observed in patients treated with ≤1 medication and those treated with ≥2 medications (Fig. 1).

### CGM metrics

We observed increases in %TIR with corresponding reductions in %TAR (>180 mg/dL, >250 mg/dL) across all therapy groups, but no significant changes in glycemic variability (Table 2). The most notable improvements in %TIR and %TAR was seen in patients treated with ≤1 medication (insulin or noninsulin). All patients maintained established %TBR targets (<4% at 70 mg/dL, <1% at <54 mg/dL) throughout the study period. Reductions in average glucose but not glycemic variability (%CV) were also observed.

**Table 2.** Changes in % Time in Range, % Time Above Range, Average Glucose and Glycemic Variability at Baseline, 3 Months, and 6 Months

Population	Baseline/month 1	Month 3	Month 6	Change month 1 vs. month 6	*P* month 1 vs. month 6
All (*n* = 38)					
%TIR (70–180 mg/dL), %, mean ± SD, (*n*)	57.0 ± 29.9 (38)	68.4 ± 24.2 (38)	72.2 ± 23.6 (38)	15.2 ± 22.3	<0.001
%TAR (>180 mg/dL) %, mean ± SD, (*n*)	43.1 ± 30.5 (33)	29.9 ± 24.9 (36)	28.2 ± 23.9 (36)	−14.9 ± 22.9	<0.001
%TAR (>250 mg/dL) %, mean ± SD, (*n*)	15.8 ± 22.9 (36)	7.5 ± 13.5 (36)	7.4 ± 11.6 (36)	−8.4 ± 16.7	0.001
Average glucose, mg/dL, mean ± SD, (*n*)	180.1 ± 47.3 (38)	160.6 ± 38.7 (38)	156.4 ± 30.8 (38)	−23.6 ± 38.8	<0.001
Glycemic variability, %CV, mean ± SD, (*n*)	24.6 ± 5.9 (35)	24.4 ± 6.0 (35)	24.4 ± 5.6 (35)	−0.2 ± 4.2	0.742
≤1 Medication (*n* = 13)					
%TIR (70–180 mg/dL), %, mean ± SD, (*n*)	62.2 ± 31.1 (13)	78.1 ± 24.4 (13)	79.8 ± 25.0 (13)	17.6 ± 16.3	0.002
%TAR (>180 mg/dL) %, mean ± SD, (*n*)	36.0 ± 32.3 (12)	19.2 ± 24.1 (12)	19.0 ± 26.0 (12)	−17.0 ± 16.9	0.005
%TAR (>250 mg/dL) %, mean ± SD, (*n*)	13.1 ± 24.8 (12)	5.2 ± 14.0 (12)	6.1 ± 13.8 (12)	−7.0 ± 14.0	0.003
Average glucose, mg/dL, mean ± SD, (*n*)	173.7 ± 50.7 (13)	142.6 ± 44.1 (13)	149.0 ± 35.9 (13)	−24.7 ± 23.9	0.003
Glycemic variability, %CV, mean ± SD, (*n*)	22.9 ± 5.9 (12)	23.3 ± 6.7 (12)	22.7 ± 5.5 (12)	−0.1 ± 4.7	0.691
≥2 Medications (*n* = 25)					
%TIR (70–180 mg/dL), %, mean ± SD, (*n*)	54.3 ± 29.5 (25)	63.4 ± 23.0 (25)	68.3 ± 22.3 (25)	13.9 ± 25.1	0.017
%TAR (>180 mg/dL) %, mean ± SD, (*n*)	46.6 ± 29.7 (24)	35.3 ± 24.0 (24)	32.7 ± 22.0 (24)	−13.9 ± 25.7	0.024
%TAR (>250 mg/dL) %, mean ± SD, (*n*)	17.2 ± 22.3 (24)	8.6 ± 13.5 (24)	8.1 ± 10.6 (24)	−9.1 ± 18.1	0.019
Average glucose, mg/dL, mean ± SD, (*n*)	183.4 ± 46.1 (25)	169.9 ± 32.7 (25)	160.3 ± 27.8 (25)	−23.1 ± 45.0	0.015
Glycemic variability, %CV, mean ± SD, (*n*)	25.5 ± 5.9 (23)	25.0 ± 5.7 (23)	25.3 ± 5.5 (23)	−0.2 ± 4.0	0.941

*P* value (two-sided) was calculated using Wilcoxon signed rank test.

TAR, time above range; TIR, time in range.

### Weight/BMI

Significant reductions from baseline to 6 months were observed in body weight (from 103.5 ± 18.2 to 100.3 ± 18.6 kg, Δ −3.1 ± 6.4 kg, *P* = 0.002) and BMI (from 35.6 ± 6.4 to 34.5 ± 6.3, Δ −1.1 ± 2.3, *P* = 0.002).

## Discussion

Results from our study demonstrated that use of rtCGM was associated with reductions in HbA1c, average glucose and glycemic variability, with increases in %TIR and corresponding decreases in %TAR in T2D patients treated with either basal insulin and/or noninsulin therapy but not prandial or premised insulin. Importantly, these improvements were achieved while patients maintained established targets for %TBR.^18^

Our findings of improved glycemic control are consistent with earlier studies of T2D patients treated with or without intensive insulin treatment.^7–9,19,20^ For example, an early study by Vigersky et al. reported significant reductions in HbA1c with intermittent rtCGM use compared with BGM in a cohort of T2D adults treated with basal insulin or noninsulin therapy.^9^

In a more recent study, investigators assessed the impact of using a novel telehealth technology/care model that combines connected rtCGM, remote lifestyle coaching, and clinical support with a mobile app.^21^ Patients were stratified by baseline HbA1c: 7.0% to <8.0%, 8.0%–9.0%, and >9.0%, At 6 months, results showed a significant improvement in HbA1c in across all baseline categories (−0.2% ± 0.8%, −0.7% ± 1.0% and −2.3% ± 1.9%, respectively, all *P* < 0.001). Participation in the program was also associated with reductions in diabetes-related distress as measured by the DDS scale, specifically in the subscale score for regimen-related distress,^22^ which is a risk factor for suboptimal glycemic control,^23,24^ increased prevalence of complications,^25,26^ all-cause mortality,^26^ and poor adherence to therapy.^27–29^

A key strength of our study was the ability to generate evidence of the effectiveness and feasibility of rtCGM use in a real-world, clinical practice setting in patients who are similar to those encountered in clinical practice. Although RCTs are necessary to provide information about the efficacy and safety of a given intervention within a controlled environment, real-world, prospective studies provide valuable information about the usage and potential benefits of rtCGM in daily diabetes management. Increasing numbers of health insurers (government and commercial) and regulatory agencies are now requiring real-world evidence in addition to RCT data when evaluating new medications and devices.^30–34^

Several limitations are notable. Because data obtained during the first month served as our baseline measures for the rtCGM metrics, our findings do not accurately assess the actual changes that occurred relative to glycemic status before rtCGM initiation. For example, it is likely that some degree of glycemic improvement occurred during the first 30 days of rtCGM use. This may account for the mismatch between the mean baseline HbA1c of 10.1% and %TIR of 57% during month 1. Therefore, the improvements reported at 3 and 6 months may underrepresent the actual improvements achieved if these metrics had been obtained (e.g., blinded CGM) before initiating unblinded rtCGM. Because we used a single-arm study design, the lack of a control arm prevents us from assessing the incremental benefits of rtCGM compared with traditional BGM.

In addition, given the diversity of our study population in terms of baseline HbA1c and therapy, we cannot identify any predominant therapeutic intervention and/or lifestyle change that impacted glycemic outcomes. For example, in some cases, medication therapy was intensified, whereas other patients may have been switched to a different or deintensified regimen. Moreover, our findings cannot be generalized to the broader T2D population given that our cohort was predominantly Caucasian. Selection bias further limits the generalizability of our results.

Finally, due to the 6-month duration of the study, the sustainability of the glycemic effects and persistence in rtCGM use remain unknown. Nevertheless, our findings provide compelling support for modifying eligibility criteria for rtCGM coverage to include T2D patients regardless of their treatment regimen or history of BGM frequency.

Current eligibility criteria established by government and commercial health plans are medically unfounded and deny coverage for the majority of T2D patients who could potentially improve their glycemic control through rtCGM use. As noted in a recent article by Anderson et al.,^16^ evidence from large RCTs^4,5^ and retrospective database^35^ studies and retrospective analyses^7^ have shown no relationship between historical frequency of daily BGM and successful use of rtCGM regardless of therapy. Regarding the requirement for intensive insulin management, a growing number of studies are demonstrating that T2D patients treated with less intensive therapy benefit from significant improvements in glycemic control.^7,9,19,20^

Although significant reductions in mean HbA1c were observed regardless of treatment (≤1 medication vs. ≥2 medications) patients treated with ≤1 medication (insulin or noninsulin) showed the greatest improvements in %TIR and %TAR without increasing hypoglycemia risk. Importantly, findings from our study challenge both the intensive insulin therapy and frequent monitoring criteria currently required for CGM coverage. As reported, all patients were performing BGM at a frequency of <3 times per day before enrollment.

In summary, rtCGM use was associated with significant glycemic improvements in T2D patients treated with basal insulin only or noninsulin therapy. Our findings call into question the relevancy of current rtCGM eligibility criteria that deny coverage to this T2D population. Eligibility requirements should be modified to expand access to rtCGM by T2D patients treated with less intensive therapies.
